# Odontogenic myxoma of the anterior mandible: Case report of a rare entity and review of the literature

**DOI:** 10.1002/ccr3.4609

**Published:** 2021-08-21

**Authors:** Maryam Sohrabi, Ramtin Dastgir

**Affiliations:** ^1^ Department of Oral and Maxillofacial Surgery School of Dentistry Tehran University of Medical Sciences Tehran Iran; ^2^ Faculty of Dentistry Tehran Medical Sciences Islamic Azad University Tehran Iran

**Keywords:** jaws, myxoma, neoplasms, odontogenic tumors, recurrence

## Abstract

This report intends to compare classic presentations of odontogenic myxoma in contrast to our case. We also suggest a comprehensive evaluation of lesions and strongly advocate against premature treatments before reaching a definitive diagnosis.

## Graphical abstract

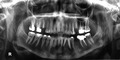

## 1 INTRODUCTION

Odontogenic myxoma is a benign but locally aggressive intraosseous lesion of the jaws. It rarely occurs in any bone other than jaws and peripherally. It originates from the mesenchymal portion of the tooth germ. It is the third most common odontogenic tumor and accounts for approximately 3%–6% of all odontogenic tumors. It has variable radiological and clinical presentations which entails thorough and prudent evaluation of the lesion. We intend to report a case of a 38‐year‐old female patient who was referred to our private practice oral and maxillofacial surgery office with chief complaint of a persistent painful lesion and teeth mobility in the anterior region of the mandible which had underwent a faulty treatment plan in an outside clinic previously. On clinical examination, the lesion revealed a diffuse and slight enlargement extending from the left mandibular first premolar to the right mandibular canine anteroposteriorly and slightly buccolingually. Overlying skin on the mental region was normal and devoid of any ulcerations, erythema, and rise in temperature. Intraoral examination did not reveal any breach in the mucosa. On radiological evaluation, orthopantomography (OPG) and cone beam computed tomography (CBCT) revealed a well‐defined, multiloculated expansile osteolytic lesion with few small septa spreading inside the lesion extending from the left mandibular first premolar to the right mandibular canine, crossing the midline. Histological evaluation confirmed the definitive diagnosis of odontogenic myxoma. The preferred surgical approach for this lesion was enucleation and curettage accompanied with peripheral ostectomy with concomitant burnishing of teeth roots. Myxomas are rare benign tumors of mesenchymal origin. They are locally invasive and occur in various tissues, including the cardiac, skeletal, cutaneous, subcutaneous tissue, aponeuroses, genitourinary tract, and skeletal muscles. It is the third most common odontogenic tumor of the jaw and most frequently occurs in the second to fifth decade of life. Women are more commonly affected than men and the mandible is the more commonly affected than maxilla. The radiographic appearances are variable as it can range from a unilocular to a multilocular radiolucency with multiple patterns of loculation, hence rendering a definitive diagnosis challenging. Treatment approaches for odontogenic myxoma remain surgical. However, it can vary from more conservative approaches like enucleation and curettage to radical resection with wide margins. This article reports an unorthodox presentation of odontogenic myxoma on account of its location within the jaw, its invasive trait, and alternating radiological features rendering it a diagnostic challenge.

The odontogenic myxoma is a painless, locally invasive but benign odontogenic neoplasm of the jaws which most frequently occurs in the second to fifth decade of life and the average age of occurrence ranges from 23 to 30 years.^1^, ^2^ It is the third most common odontogenic tumor after odontoma and ameloblastoma, respectively, and accounts for 3%–6% of all odontogenic neoplasms.^3^ There is a slight predilection for occurrence in the mandible and furthermore women are more commonly affected than men.^1^, ^4^, ^5^

Diagnosis of odontogenic myxoma is primarily based on histopathological and/or clinical and radiological findings. From a clinical point of view, odontogenic myxoma is characterized by presenting insidious and asymptomatic slow growth and expansion of the jaw bones, but with locally aggressive behavior leading to occasional cortical bone destruction, soft tissue infiltration, and dental disorders including root resorption and displacement.^6^, ^7^, ^8^, ^9^, ^10^, ^11^ Radiographically, this lesion always presents radiolucent with multiple variations. It may present as a well‐circumscribed or diffuse and unilocular or multilocular lesion. Multilocular patterns include ‘soap‐bubble’, ‘honeycomb’, and ‘tennis racket’ appearances.^2^ A ‘sun‐ray’ or ‘sun‐burst’ appearance has also been reported that may suggest a destructive, expanding behavior of this lesion.^1^ Histologically, the odontogenic myxoma is composed of haphazardly arranged stellate, spindle‐shaped, and round cells in an abundant, loose, fibrillary myxoid/mucoid stroma which contains only a few wisps of collagen fibril.^12^

Despite its benign nature, high recurrence rates have been reported, specifically after removal by curettage alone.^13^ Surgical treatment through bone resection is proposed to be the treatment of choice for this lesion.^14^

Here, we discuss a rare case of an odontogenic myxoma in an uncommon location which yielded a diagnostic challenge. Furthermore, we review the available literature, discuss differential diagnosis, and available modalities for treatment of this lesion.

## 2 CASE PRESENTATION

### 2.1 History and clinical examination

**FIGURE 1:**
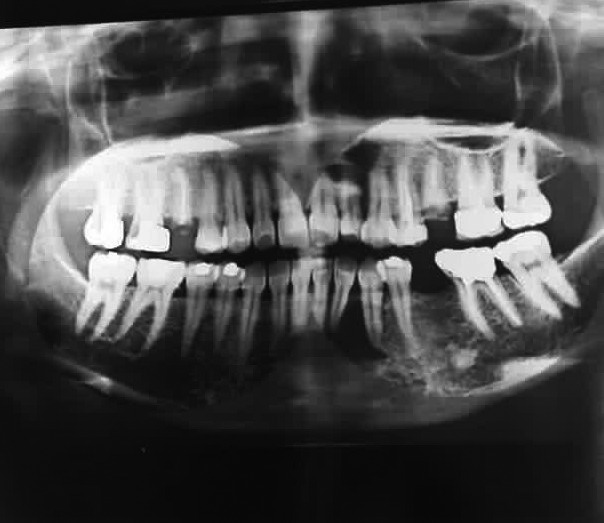
Initial orthopantomography (OPG) showing a unilocular radiolucent lesion encompassing the roots right lateral incisors and canine, strongly mimicking a radicular cyst

A 38‐year‐old female reported to an outside private dental practice office with chief complaint of pain and discomfort on her left anterior mandible region 1 year prior to presentation to our office. Initial orthopantomography (OPG) was obtained and a radiolucent lesion measuring 15 × 10 mm surrounding the roots of mandibular left lateral incisor and left canine was noticed (Figure 1). The lesion was hurriedly and erroneously diagnosed as a radicular cyst without conducting any further pulp vitality tests and the two above‐mentioned teeth were endodontically treated. A few months subsequent to the initial root canal treatment, the pain did not subside and no change in the size of the radiolucent lesion was obtained; therefore, the left mandibular central incisor was endodontically mistreated additionally without yet again conducting any further pulp vitality tests. These sequelae of mistreatments and persistent pain and discomfort following a third root canal treatment forced the patient to be referred to our oral and maxillofacial surgery private office. There were no significant issues in the patient's medical history upon presentation.

Clinical examination revealed a mild, diffuse enlargement extending from the left mandibular first premolar to the right mandibular canine anteroposteriorly and slightly buccolingually. Overlying skin on the mental region was normal and devoid of any ulcerations, erythema, and rise in temperature. Intraoral examination did not reveal any breach in the mucosa and the integrity of it remained intact. On palpation of the oral mucosa, the enlargement was bony hard on lingual cortex but with a doughy consistency on buccal plate suggesting destruction of the buccal cortical plate, diffuse, and non‐tender. Moderate mobility of five teeth including mandibular right lateral incisor to left canine was evident on palpation.

### 2.2 Investigation and differential diagnosis

**FIGURE 2:**
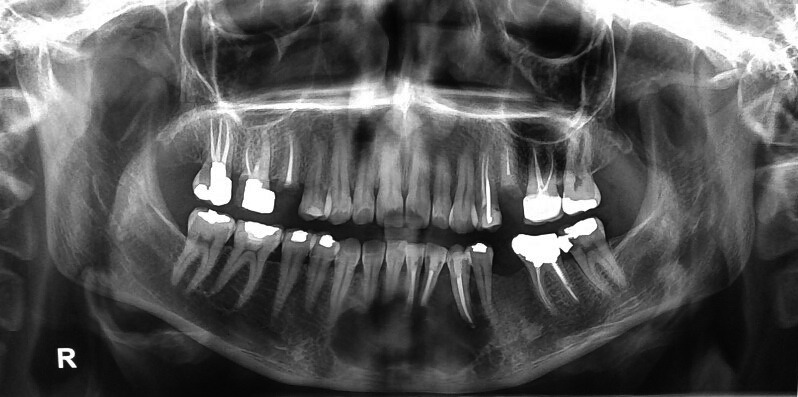
Initial orthopantomography (OPG) obtained from the patient upon presentation to our dental office. Notice the extension and multilocular pattern of radiolucency

**FIGURE 3:**
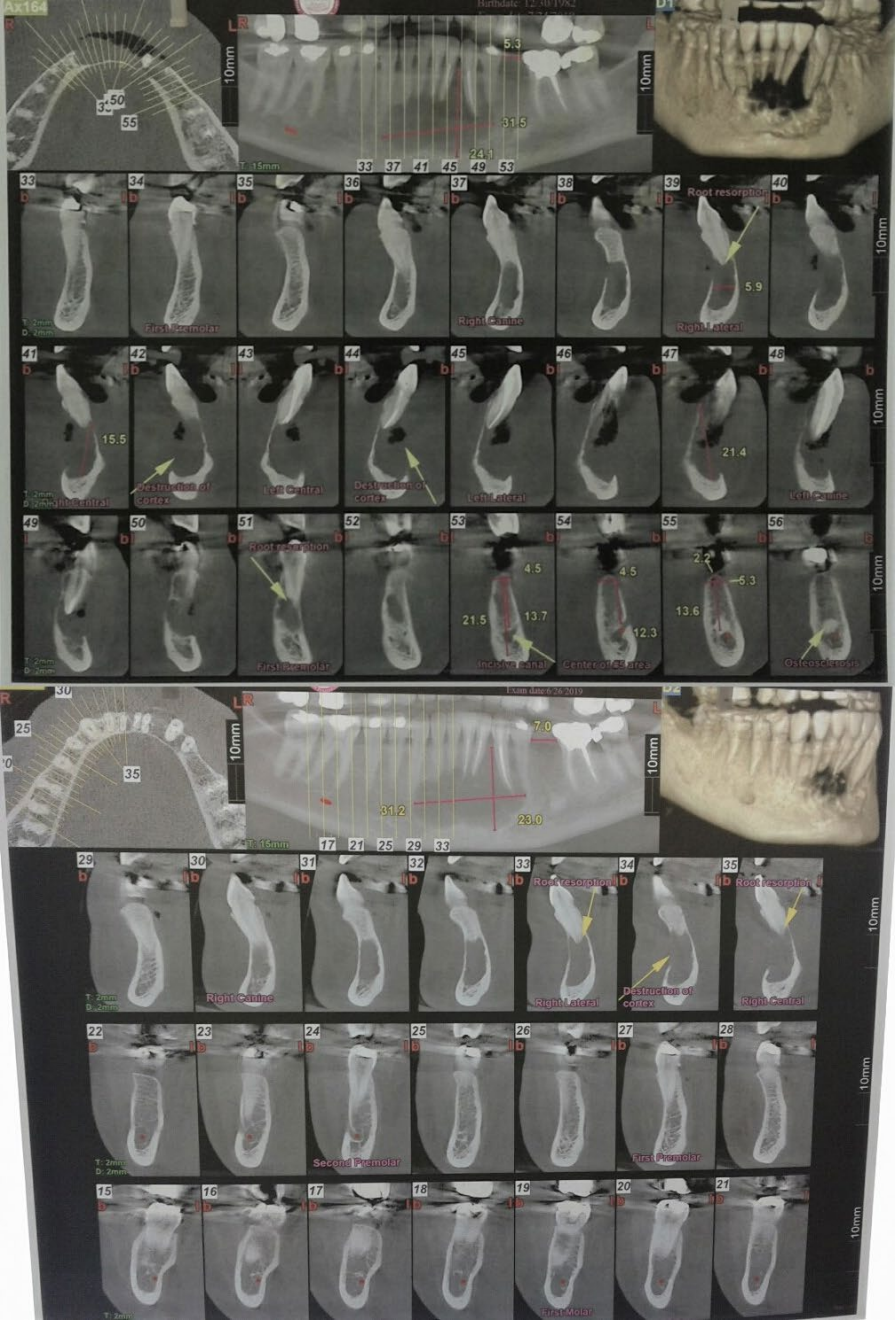
Cone beam computed tomography (CBCT) of the anterior mandible revealing the extent of the lesion. Notice the evident buccal cortex destruction

Orthopantomography (OPG) and cone beam computed tomography (CBCT) were promptly ordered. They depicted a well‐defined, multiloculated expansile osteolytic lesion with few small septa spreading inside the lesion. Expansion of the buccal cortex with areas of cortical destruction and root resorption of mandibular left central incisor, right central, and lateral incisors were also noticed. The lesion measured 31.2 × 21.0 mm extending from the left mandibular first premolar to the right mandibular canine (Figures 2 and 3). Radiographic preliminary differential diagnosis included central giant‐cell granuloma (CGCG), odontogenic keratocyst (OKC), ameloblastoma, odontogenic myxoma, and fibroameloblastoma.

**FIGURE 4:**
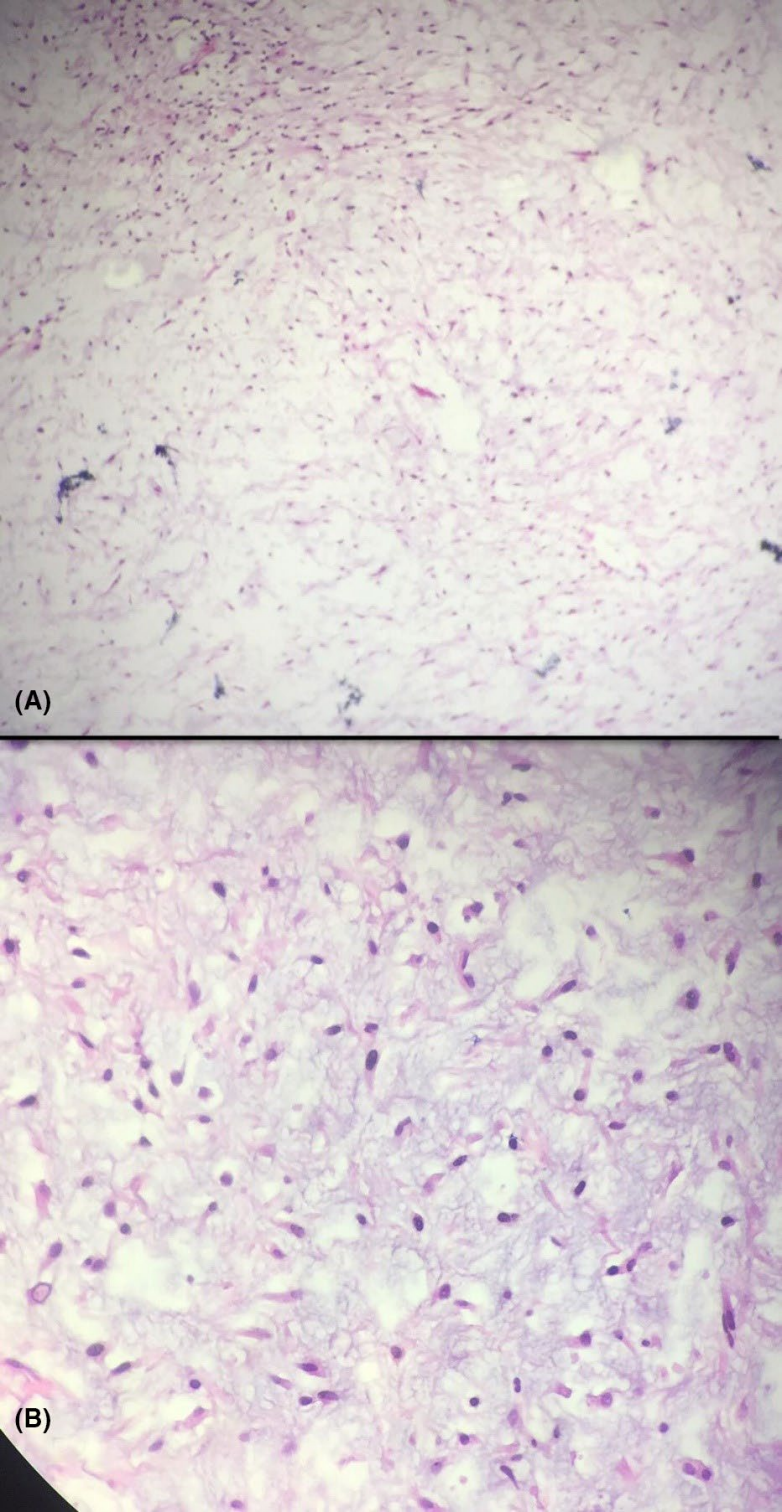
(A) Histological analysis of the excised lesion depicting an abundant loose myxoid stroma containing few collagen fibers (×20 magnification, H&E staining). (B) presence of a neo‐formed tissue composed of haphazardly arranged stellate and spindle cells with no atypia in an abundant loose myxoid stroma (×100 magnification, H&E staining)

An incisional biopsy was then conducted and the histopathological specimen showed an irregular piece of soft tan‐pink tissue with fragile consistency. Cut sections demonstrated homogenous myxoid pink surface containing hemorrhagic foci. The microscopic analysis revealed striated muscle fibers, some reactive new and pre‐existing bone trabecula and presence of a neo‐formed tissue composed of haphazardly arranged stellate and spindle cells with no atypia in an abundant loose myxoid stroma containing few collagen fibrils which was consistent with histopathological features of odontogenic myxoma and therefore a definitive diagnosis was made (Figure 4).

### 2.3 Surgical intervention

**FIGURE 5:**
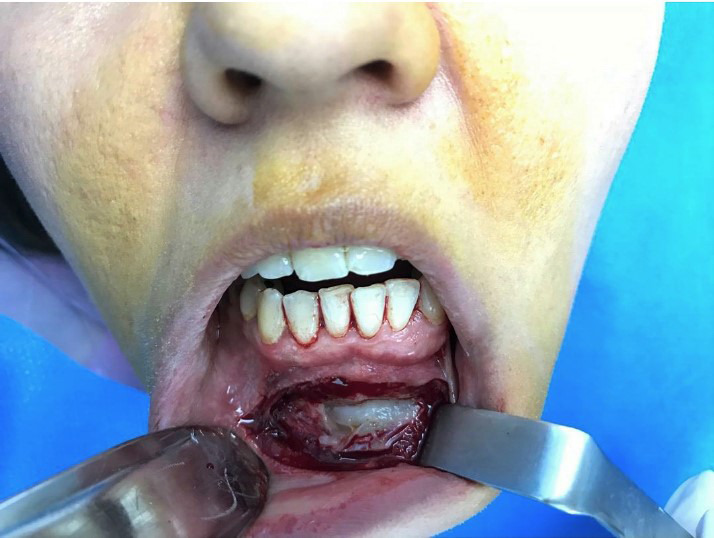
Elevation of the flap revealed destruction of the buccal cortex of the mandible. Tan‐pink tissue of the lesion is evident

**FIGURE 6:**
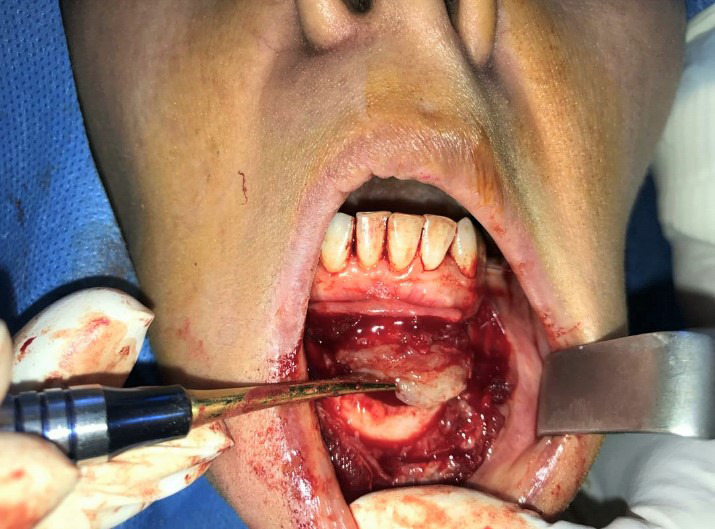
Excision of the lesion mass with curette and periosteal elevator in a piece by piece manner

**FIGURE 7:**
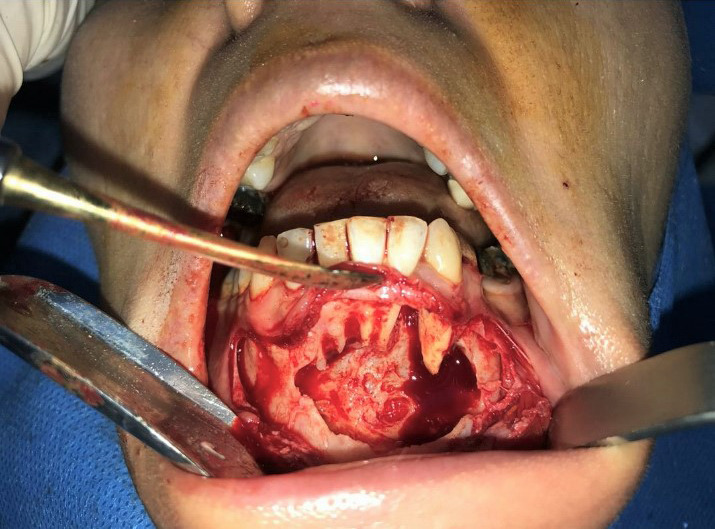
Peripheral ostectomy with a margin of 2–8 mm was conducted. Teeth roots were then cautiously burnished

**FIGURE 8:**
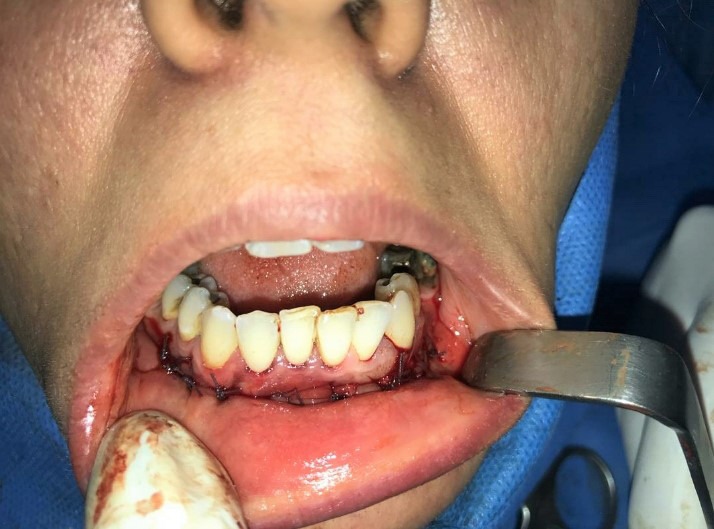
Reapproximation and suturing of the flaps

Given the patient's age, noticeable size of the lesion, its invasive and recurrent characteristics, accompanied with buccal cortex expansion and destruction, enucleation and curettage, and peripheral ostectomy accompanied with concomitant burnishing of teeth roots was planned. After obtaining a written informed consent form from the patient and informing her regarding the poor prognosis of the remaining affected teeth, the surgery was performed under local anesthesia. Surgical procedure incorporated bilateral mental nerve anesthesia, followed by a full‐thickness periosteal flap elevation with great vigilance in exposing and preserving bilateral mental nerves and subsequent excision of the lesion mass with curette and periosteal elevator in a piece by piece manner with extreme caution to preserve teeth roots (Figures 5 and 6). Teeth roots were then cautiously burnished. Afterward, peripheral ostectomy with a wide margin of 2–8 mm was conducted and the flap was reapproximated and sutured (Figures 7 and 8).

The excised specimen measuring at 2.5 × 2 × 1.5 cm accompanied with six irregular fragments of the same tissue and small bone particles aggregating to 2 × 1 × 1 cm was then placed in formalin and sent to pathology laboratory which further confirmed the definitive diagnosis of odontogenic myxoma.

The patient was informed of the recurrence tendency of the lesion and was advised for annual follow‐ups for at least 5 years.

### 2.4 Follow‐up

**FIGURE 9:**
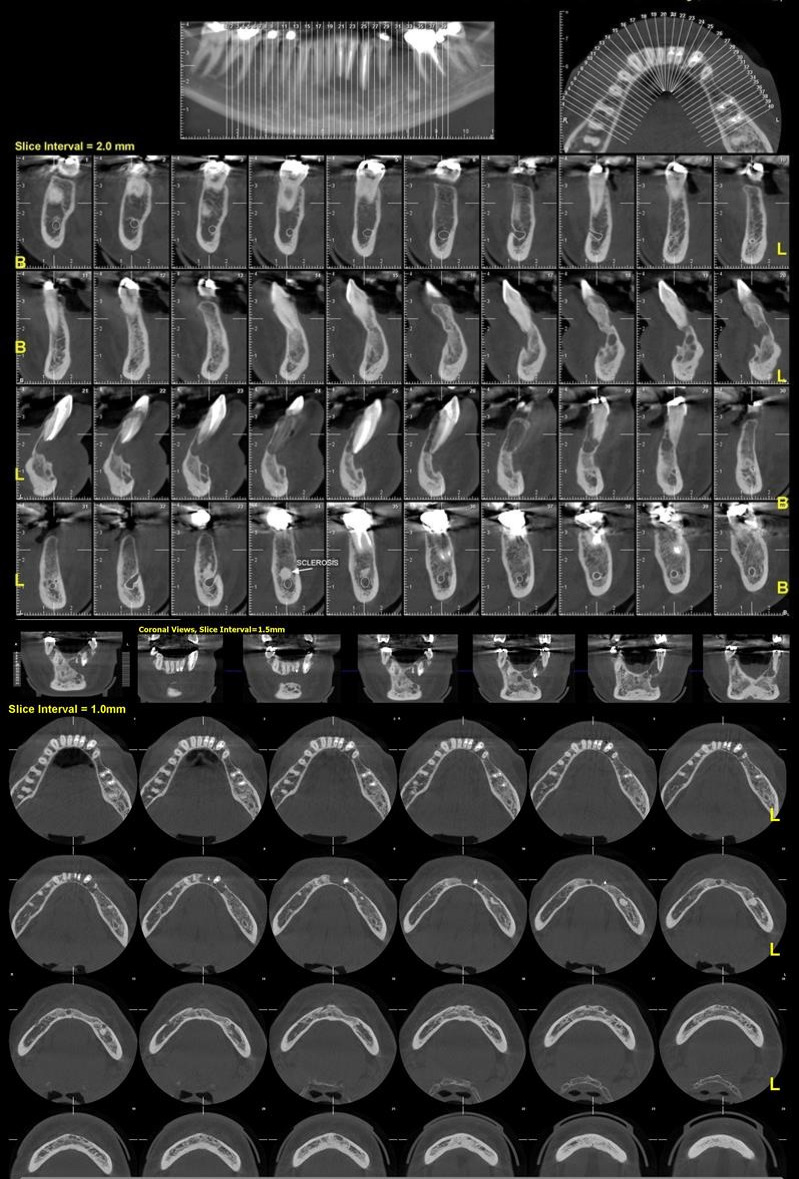
One‐year postoperative follow‐up cone beam computed tomography (CBCT) scan revealing no traces of recurrence

**FIGURE 10:**
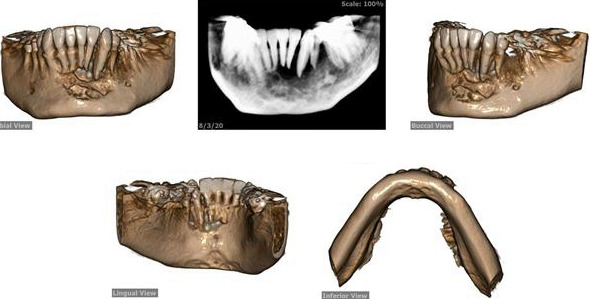
Three‐dimensional cone beam computed tomography (CBCT) scan on 1‐year follow‐up revealing no traces of recurrence

The patient's first‐ and second‐year follow‐up clinical and radiological examination revealed no recurrence of the lesion (Figures 9 and 10).

## 3 DISCUSSION AND A REVIEW OF LITERATURE

In a newly devised classification system by Bajpai et al.^15^ based on the predominance of the areas of myxoid degeneration in histopathology of the tumors of oral cavity including adipose, neural, fibroblastic, chondroblastic, muscle, odontogenic and miscellaneous tumors, odontogenic myxomas fall in the category of odontogenic tumors. Myxomas are rare benign tumors of mesenchymal origin. They are locally invasive and occur in various tissues, including cardiac, skeletal, cutaneous, and subcutaneous tissue, aponeuroses, genitourinary tract, and skeletal muscles.^16^ Odontogenic myxoma was initially named as ‘myxofibroma’ by Rudolf Virchow in 1863 due to its histologic similarity to the mucinous substance present in the umbilical cord.^17^ Later in 1947, it was renamed to ‘odontogenic myxoma’ by Thomas and Goldman.^18^ World Health Organization (WHO) defines this tumor as ‘a locally invasive neoplasm that consists of angular and rounded cells in mucoid background’.^19^

It is the third most common odontogenic tumor after odontoma and ameloblastoma, respectively, and accounts for 3%–6% of all odontogenic neoplasms.^3^ Ameloblastoma seems to be more common in Asian and African nations, whereas in North America, the most frequently diagnosed odontogenic tumor was odontome.^20^ It most frequently occurs in the second to fifth decade of life and the average age of occurrence ranges from 23 to 30 years.^1^, ^2^ Women are more commonly affected than men with a ratio of 1.5:1. The mandible is the more commonly affected than maxilla, with the posterior body, ramus, and angle being the most common sites, respectively.^5^ Regardless of the jaw, odontogenic myxoma is usually found in relation to a tooth, typically a premolar or molar.^7^ However, there have been a few new case reports of a peripheral odontogenic myxoma occurring solely on the soft tissue with no effect on the underlying bone.^21^, ^22^, ^23^, ^24^ In an updated analysis of 1692 cases by Chrcanovic et al.^25^ approximately 75% of the lesions showed signs of cortical bone perforation, 62.9% of the lesions had a radiological multilocular appearance, and 34.7% of the lesions showed the presence of angular septa. Nearly 20% of the lesions presented root resorption of adjacent teeth and 53.8% of the cases showed tooth displacement and/or uneruption due to lesion's growth. Only 6.8% of these lesions crossed the midline of the jaws which makes our case truly a rare entity.

Clinically, odontogenic myxoma is a painless, slow‐growing, benign but locally aggressive lesion that displaces and/or resorbs its adjacent structures including teeth roots and cortical bone. This lesion can advance into paranasal sinuses and zygomatic process of the maxilla as well.^5^, ^17^, ^26^ Unorthodox cases of rapidly expanding odontogenic myxoma of the jaw have been reported.^27^, ^28^ In many cases, however, these lesions are coincidentally diagnosed during a routine dental checkup.^7^, ^29^ Some odontogenic myxomas are found to be associated with unerupted teeth.^30^ Ulceration of the overlying mucosa is rarely seen and only occurs in case of the lesion being in the pathway of dental occlusion. Nevertheless, rapid growth and invasion of the soft tissue may occur as well.^31^ Our case presented with consistent pain which was in contrast of typical clinical findings of odontogenic myxoma reported in the literature.

Radiographically, odontogenic myxoma's appearance can vary from uniloculated to multiloculated and from completely radiolucent to mixed radiolucent‐radiopaque.^7^, ^25^, ^31^, ^32^ Furthermore, the margins of this lesion have been variably described as corticated, non‐corticated, poorly defined, and diffuse.^11^, ^17^, ^26^ The multilocular appearance usually presents as ‘soap‐bubble’, ‘honeycomb’, ‘tennis racket’, ‘spiderweb’, or ‘wispy’. However, recently patterns resembling a ‘sun‐ray’ or ‘sun‐burst’ appearance have also been reported that may suggest a more destructive, expanding behavior of this lesion.^1^, ^2^, ^11^, ^26^, ^33^, ^34^ The lesion usually displaces and/or resorbs adjacent teeth roots.^5^, ^26^ In some cases, this lesion is found encapsulating an unerupted tooth.^30^ Our case initially presented with uniloculated and small radiolucency which further developed to a multiloculated lesion with small septa extending into the lesion and giving it a tennis racket appearance which is consistent with typical findings of odontogenic myxoma.

Differential diagnosis based on radiographic findings differs depending on the loculation status of the lesion. For uniloculated lesions, differential diagnosis includes but not limited to periapical cyst or granuloma, lateral periodontal cyst, simple bone cyst, or unicystic ameloblastoma. For multiloculated lesions differential diagnosis include central giant‐cell granuloma (CGCG), cherubism, multicystic ameloblastoma, intraosseous hemangioma, aneurysmal bone cyst, odontogenic keratocyst (OKC), metastatic tumor, and osteosarcoma.^1^, ^2^, ^30^, ^35^, ^36^

Histologically, the bulk of odontogenic myxoma is made up of loosely arranged, spindle‐shaped and stellate cells, many of which have long fibrillar processes that tend to intermesh with apparently inactive odontogenic epithelium scattered through the myxoid (mucous) ground substance. The loose stroma tissue is mainly made up of hyaluronic acid and chondroitin sulfate as in normal tissues, but excessive in amounts. The present cells in this stroma do not show evidence of significant neoplastic activity including pleomorphism, prominent nucleoli, or mitotic figures. Growth pattern of this lesion is differentiated from other lesions by the fact that it gradually grows by secretion of ground substance rather than cellular proliferation. The gelatinous consistency of myxoma permits the lesion to permeate through bony trabeculation leaving no clear margin, therefore, making its complete removal substantially difficult.^1^, ^2^, ^3^, ^30^, ^32^, ^37^ Findings in the histological analysis of our case were consistent with the above‐mentioned characteristics; therefore, a definitive diagnosis of odontogenic myxoma was obtained.

Treatment approaches for odontogenic myxoma remain surgical. However, it can vary from more conservative approaches like enucleation and curettage to radical resection with wide margins of 1.0–1.5 cm. Although medical management of odontogenic myxoma including chemotherapy has been utilized in few recurrence cases, its use is not advocated.^5^, ^38^ Furthermore, due to the radio‐resistant trait of odontogenic myxoma, radiotherapy also has no role in management of this lesion.^1^, ^25^, ^30^ Boffano et al.^39^ have advocated resection of myxomas larger than 3 centimeters, and enucleation and curettage of lesions smaller than that. The idea behind utilization of these rather contrasting approaches originates from the growth and permeation characteristics of odontogenic myxoma, depriving this lesion from a well‐defined border. Furthermore, radical resection not only leaves the patient with significant cosmetic and functional defects, but also it is not always successful in preventing recurrence of the lesion.^5^, ^40^ Current literature suggests that aggressive management of this lesion may not be necessary, especially as first‐line approach.^5^ Allphin et al.^41^ suggested a more conservative approach as the first‐line treatment of odontogenic myxoma, followed by respective surgery if deemed necessary. However, when radical surgery is performed, delayed reconstruction must be considered due to odontogenic myxoma's high tendency to recur.^6^ Utilization of liquid nitrogen cryotherapy has also been recently reported in adjunct to surgical modalities.^22^ In our case, enucleation and curettage accompanied with peripheral ostectomy and concomitant burnishing of teeth roots were utilized.

Odontogenic myxomas are notorious for their very high recurrence rate, consisting up to 25% following enucleation and curettage alone.^42^, ^43^ Therefore, follow‐up is recommended throughout the patient's life.^5^, ^44^ However, at minimum, a follow‐up period of 5 years is highly recommended, since this the time period where majority of recurrences occur.^6^ Our patient was recalled to the clinic for the first‐ and second‐year follow‐ups, both of which revealed no signs of recurrence.

## 4 CONCLUSION

Due to its aggressive nature, odontogenic myxoma, though benign, requires extreme levels of awareness throughout the diagnosis, treatment, and follow‐up phases. This benign odontogenic tumor can present with a wide variety of clinical and radiological depictions. As seen in our case, it presented with a unilocular radiolucency encompassing teeth roots, highly mimicking the clinical behavior of a radicular cyst. The alternating multiloculartiy and unilocularity characteristics of this lesion compels the clinician to consider numerous differential diagnosis which can only be fortified by a thorough histological analysis in order to reach a definitive diagnosis which was unfortunately lacking on the side of our patient's primary dentist, leading to unnecessary mistreatments of three endodontically sound teeth and further expansion of the lesion, leaving a larger residual defect. This report intends to compare classic presentations of odontogenic myxoma in contrast to our case in terms of unorthodox region, the fact that it crossed the midline of the jaw, and altered radiographic appearance, evolving from unilocular to multilocular pattern which occurs very rarely. We also suggest a detailed and comprehensive evaluation of lesions and strongly advocate against premature treatments before reaching a definitive diagnosis. As in our case, if proper management had been done by the patient's primary dentist, three teeth could have been salvaged and not mistakenly treated and further destruction of cortical bone could have been prevented.

## CONFLICT OF INTEREST

None declared.

## AUTHOR CONTRIBUTIONS

MS conducted the surgery. RD was responsible for data collection and writing of the manuscript. RD and MS were responsible for editing and revising of the manuscript. All authors discussed the results and contributed to the final manuscript.

## ETHICAL APPROVAL

Ethical approval by Ethics Committee of Tehran Medical Sciences, Faculty of Dentistry, Islamic Azad University.

## DECLARATION OF PATIENT CONSENT

The patient consents to their clinical findings, photographs, radiographs, and results to be reported in this article. The patient understands that their name or initials will not be mentioned anywhere in this report and due efforts will be made to completely conceal their identity, however, a total anonymity cannot be guaranteed.

## Data Availability

The data that support the findings of this study are available from the corresponding author upon reasonable request.

## References

[1] Sivapathasundharam B. Shafer’s Textbook of Oral Pathology, 9th ed. New Delhi, India: Elsevier; 2020.

[2] Regezi JA, Sciubba JJ, Jordan RCK. Oral Pathology: Clinical Pathologic Correlations, 7th ed. Philadelphia, PA: Saunders; 2016.

[3] Odell EW. Cawson’s Essentials of Oral Pathology and Oral Medicine, 9th ed. New Delhi, India: Elsevier; 2017.

[4] Gupta S, Grover N, Kadam A, Gupta S, Sah K, Sunitha JD. Odontogenic myxoma. Natl J Maxillofac Surg. 2013;4(1):81‐83. 24163558 10.4103/0975-5950.117879 PMC3800391

[5] Shupak RP, Cho JJ. Mandibular odontogenic myxoma in a paediatric patient. BMJ Case Rep. 2020;13(10):e236926. 10.1136/bcr-2020-236926 PMC7604807 33127701

[6] Leiser Y, Abu‐El‐Naaj I, Peled M. Odontogenic myxoma–a case series and review of the surgical management. J Craniomaxillofac Surg. 2009;37(4):206‐209. 19027311 10.1016/j.jcms.2008.10.001

[7] Li T‐J, Sun L‐S, Luo H‐Y. Odontogenic myxoma: a clinicopathologic study of 25 cases. Arch Pathol Lab Med. 2006;130(12):1799‐1806. 17149953 10.5858/2006-130-1799-OMACSO

[8] Francisco A‐L‐N, Chulam T‐C, Silva F‐O, et al. Clinicopathologic analysis of 14 cases of odontogenic myxoma and review of the literature. J Clin Exp Dent. 2017;9(4):e560‐e563. 28469823 10.4317/jced.52953 PMC5410678

[9] Dotta JH, Miotto LN, Spin‐Neto R, Ferrisse TM. Odontogenic myxoma: systematic review and bias analysis. Eur J Clin Invest. 2020;50(4):e13214. 32048275 10.1111/eci.13214

[10] Hammad HM, Hasen YM, Odat A‐AM, Mikdadi AM, Safadi RA. Odontogenic myxoma with diffuse calcifications: a case report and review of a rare histologic feature. Oral Surg Oral Med Oral Pathol Oral Radiol. 2016;122(4):e116‐e124. 26948020 10.1016/j.oooo.2015.12.009

[11] Noffke CEE, Raubenheimer EJ, Chabikuli NJ, Bouckaert MMR. Odontogenic myxoma: review of the literature and report of 30 cases from South Africa. Oral Surg Oral Med Oral Pathol Oral Radiol Endod. 2007;104(1):101‐109. 17507265 10.1016/j.tripleo.2007.01.026

[12] Banasser AM, Bawazir MM, Islam MN, Bhattacharyya I, Cohen DM, Fitzpatrick SG. Odontogenic myxoma: a 23‐year retrospective series of 38 cases. Head Neck Pathol. 2020;14(4):1021‐1027. 32506377 10.1007/s12105-020-01191-7 PMC7669973

[13] Higo M, Kasamatsu A, Ogawara K, Shiiba M, Uzawa K, Tanzawa H. A case of a rapidly expanding odontogenic myxoma of the mandible. Oral Sci Int. 2015;12(1):22‐26.

[14] Limdiwala P, Shah J. Odontogenic myxoma of maxilla: a review discussion with two case reports. Contemp Clin Dent. 2015;6(1):131‐136. 25684930 10.4103/0976-237X.149310 PMC4319334

[15] Bajpai M, Pardhe N. A simplified working classification proposed for myxoid tumors of oral cavity. Iran J Pathol. 2017;12(4):413‐414. 29563941 PMC5844690

[16] Kyriakos M. Tumours and tumour like conditions of the soft tissue. In: Kissane JM, ed. Anderson's Pathology, Vol 2, 9th ed. St. Louis: The CV Mosby Company; 1990:1838‐1928.

[17] Varun A, Ramachandran S, Rajasekharan A, Balan A. Odontogenic myxoma: an archetypal presentation of a rare entity. J Indian Acad Oral Med Radiol. 2016;28(4):465.

[18] Thoma KH, Goldman HM. Central myxoma of the jaw. Am J Orthod Oral Surg. 1947;33(7):B532‐B540. 10.1016/0096-6347(47)90315-3 20251479

[19] Brannon RB. Central odontogenic fibroma, myxoma (odontogenic myxoma, fibromyxoma), and central odontogenic granular cell tumor. Oral Maxillofac Surg Clin North Am. 2004;16(3):359‐374. 18088737 10.1016/j.coms.2004.03.004

[20] Mascitti M, Togni L, Troiano G, et al. Odontogenic tumours: a 25‐year epidemiological study in the Marche region of Italy. Eur Arch Otorhinolaryngol. 2020;277(2):527‐538. 31612338 10.1007/s00405-019-05683-3

[21] Kanitkar S, Kamat M, Tamagond S, Varekar A, Datar U. Peripheral odontogenic myxoma in a 12‐year‐old girl: a rare entity. J Korean Assoc Oral Maxillofac Surg. 2017;43(3):178‐181. 28770159 10.5125/jkaoms.2017.43.3.178 PMC5529192

[22] Aytac‐Yazicioglu D, Eren H, Görgün S. Peripheral odontogenic myxoma located on the maxillary gingiva: report of a case and review of the literature. Oral Maxillofac Surg. 2008;12(3):167‐171. 18642035 10.1007/s10006-008-0122-8

[23] Mascitti M, Togni L, Pirani F, Rubini C, Santarelli A. Peripheral odontogenic myxoma: report of two new cases with a critical review of the literature. Open Dent J. 2018;12(1):1079‐1090.

[24] Bajpai M, Pardhe N. Extra‐osseous odontogenic myxoma of maxillary gingiva. J Coll Physicians Surg Pak. 2017;27(3):S28‐S29. 28302237

[25] Chrcanovic BR, Gomez RS. Odontogenic myxoma: an updated analysis of 1,692 cases reported in the literature. Oral Dis. 2019;25(3):676‐683. 29683236 10.1111/odi.12875

[26] Shivashankara C, Nidoni M, Patil S, Shashikala KT. Odontogenic myxoma: a review with report of an uncommon case with recurrence in the mandible of a teenage male. Saudi Dent J. 2017;29(3):93‐101. 28725126 10.1016/j.sdentj.2017.02.003 PMC5503096

[27] Moussa A, Achach T, Njim L, Yahia NB, Gassab E, Zakhama A. Odontogenic myxoma: a report of an unusual pediatric case. Int J Pediatr Otorhinolaryngol Extra. 2007;2(3):173‐175.

[28] Hirai E, Yamamoto K, Yonemasu H, Takahashi O, Takao M, Fushimi C. Odontogenic myxoma containing osteocement‐like tissue: report of a case with an unusual histopathological feature. J Oral Maxillofac Surg Med Pathol. 2014;26(3):407‐410.

[29] Benjelloun L, Cherradi N, Kessab A, Dghoughi S. An atypical odontogenic myxoma. J Stomatol Oral Maxillofac Surg. 2018;119(2):154‐157. 29229314 10.1016/j.jormas.2017.12.001

[30] Bisla S, Gupta A, Narwal A, Singh V. Odontogenic myxoma: ambiguous pathology of anterior maxilla. BMJ Case Rep. 2020;13(8):e234933. 10.1136/bcr-2020-234933 PMC7449536 32843449

[31] Kaffe I, Naor H, Buchner A. Clinical and radiological features of odontogenic myxoma of the jaws. Dentomaxillofac Radiol. 1997;26(5):299‐303. 9482003 10.1038/sj.dmfr.4600261

[32] Altug HA, Gulses A, Sencimen M. Clinico‐radiographic examination of odontogenic myxoma with displacement of Unerupted upper third molar: review of the literature. Int J Morphol. 2011;29:930‐933.

[33] Moore BA, Wine T, Burkey BB, Amedee RG, Butcher RB 2nd. Sphenoid sinus myxoma: case report and literature review. Ochsner J. 2008;8(4):166‐171. 21603497 PMC3096368

[34] White SC, Pharoah MJ. Oral Radiology: Principles and Interpretation, 7th ed. St. Louis, MO: Mosby; 2014.

[35] Wood NK, Goaz PW. Differential Diagnosis of Oral and Maxillofacial Lesions, 5th ed. St. Louis, MO: Mosby; 1997.

[36] Chi AC, Neville BW, Damm DD, Allen CM. Oral and Maxillofacial Pathology, 4th ed. Philadelphia, PA: Saunders; 2017.

[37] Zhang J, Wang H, He X, Niu Y, Li X. Radiographic examination of 41 cases of odontogenic myxomas on the basis of conventional radiographs. Dentomaxillofac Radiol. 2007;36(3):160‐167. 17463101 10.1259/dmfr/38484807

[38] Kleiber GM, Skapek SX, Lingen M, Reid RR. Odontogenic myxoma of the face: mimicry of cherubism. J Oral Maxillofac Surg. 2014;72(11):2186‐2191. 25200927 10.1016/j.joms.2014.05.027

[39] Boffano P, Gallesio C, Barreca A, Bianchi FA, Garzino‐Demo P, Roccia F. Surgical treatment of odontogenic myxoma. J Craniofac Surg. 2011;22(3):982‐987. 21558907 10.1097/SCS.0b013e3182101400

[40] Ogütcen‐Toller M, Sener I, Kasap V, Cakir‐Ozkan N. Maxillary myxoma: surgical treatment and reconstruction with buccal fat pad flap: a case report. J Contemp Dent Pract. 2006;7(1):107‐116. 16491153

[41] Allphin AL, Manigilia AJ, Gregor RT, Sawyer R. Myxomas of the mandible and maxilla. Ear Nose Throat J. 1993;72(4):280‐284. 8486107

[42] McFarland M, Abaza NA, El‐Mofty S. Mouth, teeth and pharynx. In: Damjanov I, Linder J, eds. Anderson’s Pathology, Vol 2, 10th ed. St Louis: Mosby; 1996:1563‐1615.

[43] Speight PM. Tumours of oral cavity. In: Fletcher CDM, ed. Diagnostic Histopathology of Tumors, Vol 1, 4th ed. Philadelphia: Elsevier Saunders; 2013:246‐269.

[44] Muzio LL, Nocini P, Favia G, Procaccini M, Mignogna MD. Odontogenic myxoma of the jaws A clinical, radiologic, immunohistochemical, and ultrastructural study. Oral Surg Oral Med Oral Pathol Oral Radiol Endod. 1996;82(4):426‐433. 8899782 10.1016/s1079-2104(96)80309-x

